# Rhabdomyosarcomatous Transformation of a Gastrointestinal Stromal Tumor following Treatment with Imatinib

**DOI:** 10.1155/2015/317493

**Published:** 2015-01-28

**Authors:** Xiaoyin Jiang, H. Bryan Anderson, Cynthia D. Guy, Paul J. Mosca, Richard F. Riedel, Diana M. Cardona

**Affiliations:** ^1^Department of Pathology, Duke University, DUMC Box 3712, Durham, NC 27710, USA; ^2^St. George's University, St. George, Grenada; ^3^Department of Surgery, Duke University, 3116 North Duke Street, Durham, NC 27704, USA; ^4^Department of Medicine, Duke University, DUMC Box 3198, Durham, NC 27710, USA

## Abstract

Rhabdomyosarcomatous dedifferentiation of GIST following tyrosine kinase inhibitor (TKI) therapy is rare, with only a handful of cases previously reported in the literature. Herein we present a case of metastatic GIST initially treated with imatinib that developed radiographic evidence of progression after 8 months of standard dose therapy with continued progression despite attempts at using dose-escalated imatinib 400 mg bid. Due to the patient's worsening clinical symptoms and radiographic concerns for colonic thickening, abscess, and extraluminal air, the patient underwent a palliative resection of a large heterogeneous mass arising from the posterior stomach and several metastatic foci. Pathology revealed a dedifferentiated GIST with rhabdomyosarcomatous features. This report will highlight the unique features of this case and review the existing literature.

## 1. Introduction

Gastrointestinal stromal tumors (GISTs) are the most common mesenchymal tumors of the alimentary tract. GISTs most commonly affect adults over the age of 50 with a slight male predominance [1]. These neoplasms are believed to arise from the interstitial cells of Cajal and over 80% express CD117 (c-Kit) by immunohistochemistry (IHC) [2]. Characteristically these neoplasms contain activating mutations in KIT, or less commonly platelet derived growth factor receptor alpha (PDGFRA). These genetic alterations result in a gain of function or constitutive activation of the encoded tyrosine kinases [1]. Morphologically, GISTs are composed of spindle, epithelioid, or rarely pleomorphic cells and most commonly also express CD34 and DOG1 antigens by IHC [2, 3]. Interestingly, relatively recent reports of rhabdoid or rhabdomyosarcomatous (RMS) differentiation have been described in these tumors [4–6].

Imatinib, a tyrosine kinase inhibitor (TKI), is the current mainstay of treatment for individuals with unresectable or metastatic disease based on data from Demetri et al. showing sustained objective response in more than half of patients treated [7]. This potential drug response is most efficacious in those tumor harboring exon 11 Kit mutations, while those with exon 9 mutations showed worse prognosis and benefited more from higher-dose therapy [8].

Herein we report a case of metastatic GIST with rhabdomyosarcomatous transformation following treatment with imatinib.

## 2. Materials and Methods

Surgical specimens were fixed in 10% neutral buffered formalin. Following fixation gross examination was performed and representative sections were embedded in paraffin. Five micron thick hematoxylin and eosin stained sections were created. Immunohistochemistry for CD117 (rabbit monoclonal, Cell Marque, Rocklin, CA), CD34 (clone MY10, BD Biosciences, San Jose, CA), DOG1 (clone K9, Leica Biosystems, Buffalo Grove, IL), desmin (clone DE-R-11, Leica Biosystems), smooth muscle actin (clone alpha sm-1, Leica Biosystems), myoD1 (clone 5.8A, Dako, Carpinteria, CA), and myogenin (clone F5D, Dako) was performed. Molecular analysis for KIT mutation was performed at an outside laboratory (OHSU, Portland, OR) using DNA extraction and purification of paraffin embedded tumor tissue.

## 3. Results

### 3.1. Clinical History

**Figure 1:**
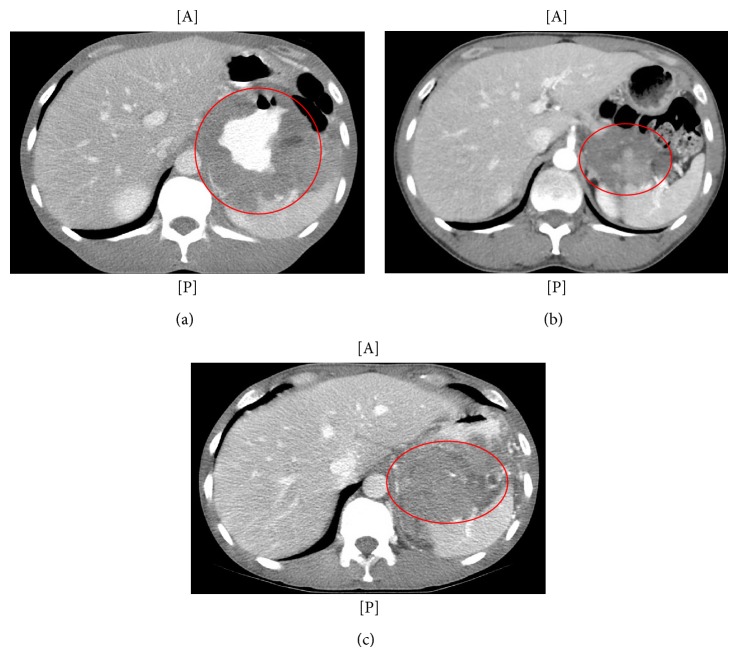
CT scans from three time points showing a gastric mass. (a) Initial presentation. (b) Following 5 months of imatinib therapy. Note tumor response as compared to (a). (c) Following 10 months of imatinib therapy. Note tumor progression (tumor circled).

A 47-year-old African American male presented to the emergency department with complaints of right lower quadrant abdominal pain and a 20-pound weight loss over the prior two months. The patient had no significant past medical history or any other symptomatology. Computerized tomography (CT) imaging revealed a 14 cm tumor with possible central necrosis that originated from the posterior gastric wall and extended superiorly to the diaphragm (Figure 1(a)). Additionally, there appeared to be metastases in the right pelvic cavity (5.5 cm) and within a right inguinal hernia (4.5 cm). An endoscopic biopsy of the gastric lesion revealed a spindle cell neoplasm which was strongly and diffusely immunoreactive for CD117, CD34, and DOG1. S-100 protein, smooth muscle actin, desmin, and cyokeratin IHC were negative. The diagnosis of GIST was rendered. The patient was initiated on imatinib 400 mg daily. Initial molecular testing was negative for exon 9 or exon 11 mutations. Two months after initiation of treatment, however, there was radiographic evidence of treatment response with a significant decrease in size of all tumors (Figure 1(b)).

Eight months after initiation of imatinib CT imaging demonstrated tumor regrowth and heterogeneous enhancement at the primary tumor site while other metastatic sites remained stable. The dose of imatinib was subsequently escalated to 800 mg daily (400 mg bid).

Approximately one year after his initial presentation, the patient presented with upper gastrointestinal bleeding and an associated microperforation due to tumor progression (Figure 1(c)). Given concerns for abscess and developing fistula by imaging, a palliative surgical procedure was undertaken and included en bloc resection of the tumor with a total gastrectomy/Roux-en-Y esophagojejunostomy, distal pancreatectomy, splenectomy, left partial hepatectomy, and extended right colectomy.

### 3.2. Histopathological Diagnosis and Genetic Analysis

**Figure 2:**
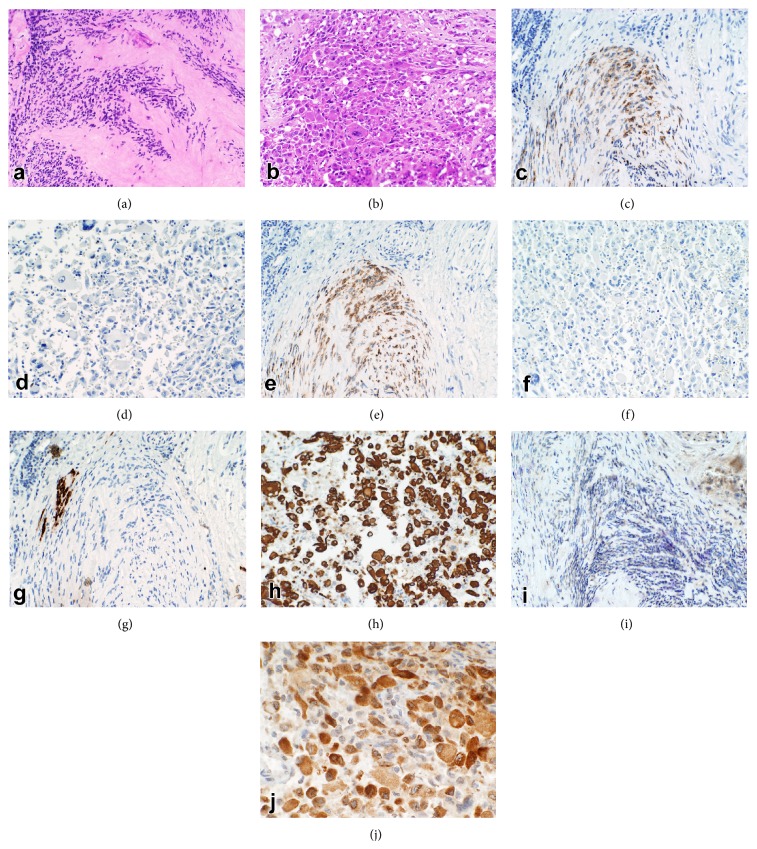
Tumor at the time of en bloc resection. Left panels showing H&E (a, b) and immunophenotype of spindle cell component (a, c, e, g, and i) and right panels showing rhabdomyosarcomatous component (b, d, f, h, and j) as follows: (c, d) c-kit IHC, 200x, (e, f) DOG1 IHC, 200x, (g, h) desmin IHC, 200x, and (i, j) myoD-1, 400x.

The resection revealed a 10.4 × 6.4 × 6.3 cm tumor arising from the stomach and invading into the spleen and pancreas. The bulk of the tumor was composed of pleomorphic, eosinophilic polygonal cells with bizarre nuclei, abundant cytoplasm, and increased mitotic activity (up to 3 mitotic figures per 50 high power fields) (Figure 2(b)). Also observed were areas of marked hyalinization, consistent with treatment effect, and focal areas (<5% of the lesion) of a spindle cell tumor reminiscent of his previously biopsied GIST (Figure 2(a)). The pleomorphic tumor cells were strongly positive for desmin and focally positive for myoD1 but negative for myogen (Myf-4), CD117, CD34, and DOG1 IHC, suggesting rhabdomyosarcomatous differentiation. Inversely, the spindle cell component was positive for CD117, DOG1, and CD34 and negative for the RMS antigens by IHC (Figures 2(c)–2(j)). A diagnosis of a dedifferentiated GIST with rhabdomyosarcomatous differentiation was made. Molecular testing for KIT mutations was performed on the two distinct histologic areas and both revealed an exon 11 deletion KV558-559. No imatinib resistance mutations were detected in exon 13, 17, or 18. Additionally, multiple fibrotic omental nodules (up to 1.4 cm) were resected at this time. These were morphologically consistent with GISTs with marked tumor response to imatinib treatment. No rhabdomyosarcomatous differentiation was seen in these separate tumors. The patient was reinitiated on imatinib 400 mg daily and has remained on that dose for the past 4 years with evidence of stable disease.

## 4. Discussion

To our knowledge, only 6 cases of rhabdomyosarcomatous dedifferentiation in GISTs have been reported following treatment with TKIs [4, 5]. Heterologous differentiation of the primary tumor (as opposed to the metastases) has only been reported in 1 other case. This is the first report of the exon 11 deletion KV558-559 in this clinical setting.

The rare cases of dedifferentiated tumors have generally demonstrated a more aggressive clinical course, with recurrence and metastases. This stands in contrast to primary GISTs with rhabdoid morphology, which, although also uncommon, have not demonstrated malignant behavior [6, 9].

Based on the small number of previously reported cases, dedifferentiated tumors, as in our case, demonstrate resistance to the currently available TKI therapy. Time to treatment failure in these rare tumors with RMS dedifferentiation ranged from 10 to 24 months [4, 5]. Our patient had only 8 months of stable disease following TKI therapy.

In general, when treatment failure occurs, a unique pattern of disease progression follows, termed a “resistant nodule.” These nodules are seen on imaging as new central or peripheral areas of enhancement within preexisting responding lesions. These imatinib “resistant nodules” appear in about 50% of patients after two years of therapy and are frequently found to have novel mutations in KIT or PDGFR (that were not present in the primary tumor). These findings support the hypothesis that “resistant nodules” arise through clonal evolution [10]. However, of the reported GISTs with RMS dedifferentiation, only 1 of 6 has shown secondary KIT or PDGFR mutations [4]. The remaining five, as in our case, have only shown primary-type exon 11 mutations. This has led to the proposition that the dedifferentiation may be related to an alternative mechanism for TKI resistance, the genetic basis of which has yet to be established.

Curiously, the initial biopsy in our case was negative for KIT and PDGFRA mutations. This possibly represents a false negative result as the tumor was initially objectively responsive to therapy, and tumors have not previously been reported to gain a primary-type (exon 9 or 11) mutation upon progression.

With regard to therapy, sunitinib and regorafenib are approved therapies in the second and third lines, respectively, but both have demonstrated only limited success [11–13]. Novel adjunct therapies targeting protein products downstream of KIT, such as PI3-K and MAPK/MEK, have been suggested, but to date these therapies have not become widely accepted or available [14]. Insufficient data exists on whether standard rhabdomyosarcoma treatments such as dactinomycin, vincristine, and cyclophosphamide would be more beneficial than TKIs for patients with dedifferentiated GISTs.

## 5. Conclusions

In summary, we report a genetically unique case of rhabdomyosarcomatous dedifferentiation in a GIST following TKI therapy. Our case supports prior reports that these tumors behave in an aggressive fashion with early recurrence and resistance to TKI treatment. The molecular findings suggest that the resistance to TKI therapy in these rare tumors is driven more by alternative mechanisms than through secondary Kit/PDGFRA mutations. Further study is needed to clarify these mechanisms as well as determine optimal treatment strategies.
